# The impact of preoperative parameters on postoperative foveal displacement in idiopathic macular hole

**DOI:** 10.1038/s41598-024-54417-x

**Published:** 2024-02-14

**Authors:** Hecong Qin, Jinsong Zhao, Siyan Jin, Hui Zhang

**Affiliations:** Department of Ophthalmology, The Second Hospital of Jilin University, Changchun City, China

**Keywords:** Diseases, Medical research

## Abstract

This study examined the effect of vitrectomy combined with internal limiting membrane (ILM) peeling on foveal displacement in 42 eyes with idiopathic macular hole (IMH). A retrospective analysis was conducted to measure various macular hole parameters before surgery, including basal diameter, minimum diameter, hole height, and areas affected by traction such as macular hole area (MHA), macular hole cystoid space area (MHCSA), macular hole retinal area (MHRA), and total area (TA). The results showed a postoperative shift of the fovea towards the optic disc in all cases. Notably, the extent of foveal displacement was significantly linked to the preoperative basal diameter (*r*_*s*_ = 0.405, *P* = 0.008) but not to other preoperative parameters or postoperative visual acuity. Furthermore, the study found that the temporal side of the macular hole was more affected by traction than the nasal side preoperatively, leading to greater postoperative displacement (All *P* < 0.05).

## Introduction

Idiopathic macular hole (IMH) is a common form of vitreoretinal interface disease, primarily treated with Pars plana vitrectomy (PPV) combined with internal limiting membrane (ILM) peeling^1–3^. This surgical approach is aimed at relieving the traction force around the hole, significantly improving hole closure rates and reducing postoperative re-openings from 7.12 to 1.18%^4,5^. Widely adopted since its introduction in 1997, this technique has demonstrated effectiveness in promoting recovery^6^. However, research by Kawano et al. in 2013 indicated a postoperative shortening of the distance between the macula and optic disc after ILM peeling, suggesting foveal displacement towards the optic disc^7^. This type of retinal displacement, distinct from the overall positional changes seen after retinal detachment surgery^8^, is attributed to the increased flexibility of the macular retina post-ILM peeling and the constant traction of the optic disc^9^. Despite the phenomenon of macular displacement has garnered significant attention from researchers over the past decade^10,11^, further studies are needed to fully understand the morphological changes and underlying mechanisms of this phenomenon.

Presently, research on postoperative displacement after IMH treatment predominantly focuses on shifts in the macular fovea position following successful hole closure. Although various predictors for hole closure, such as basal diameter, macular hole index, and traction index, have been proposed, these primarily reflect single-direction pulling forces near the hole^12^. This novel approach, focusing on the area of preoperative traction, has shown greater sensitivity in predicting outcomes than traditional linear measurements^13^. Notably, macular displacement typically occurs within the first two weeks post-surgery, a period coinciding with hole closure^10,14^, hinting at a possible link between movement and the closure process or preoperative morphology^7^. This study aims to retrospectively analyze postoperative macular displacement in IMH patients treated with ILM peeling, incorporating area parameters to explore the relationship between preoperative traction areas and postoperative displacement. Our findings will further advance the exploration of the mechanisms and significance of foveal displacement.

## Result

### Patient characteristics

**Figure 1:**
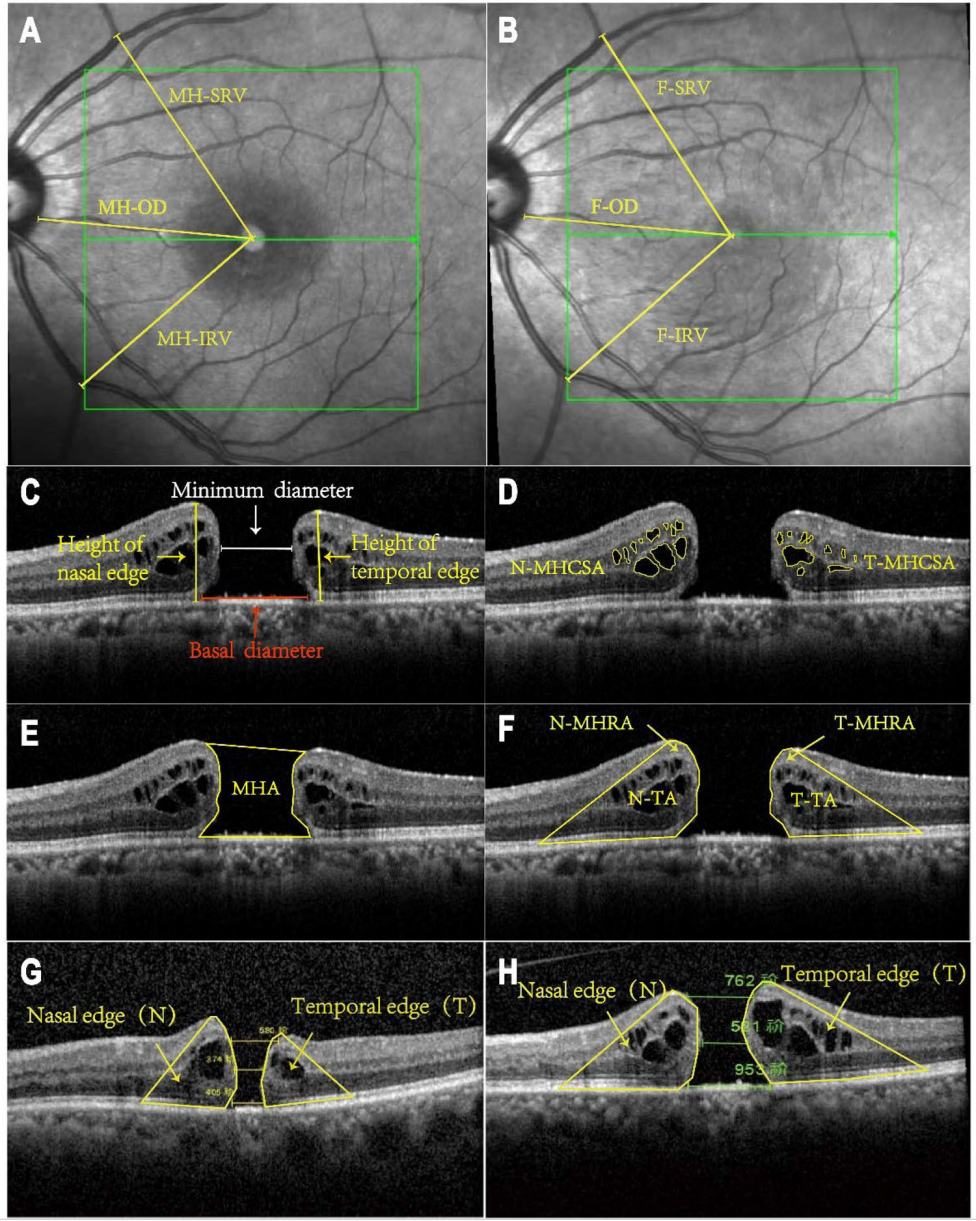
(**A**) MH-OD: The distance between the center of the hole and the optic disc margin. MH-SRV: The distance between the center of the hole and the superior retina vascular bifurcation.MH-IRV: The distance between the center of the hole and the inferior retina vascular bifurcation. (**B**) F-OD, F-SRV, and F-IRV represented the postoperative distances between the fovea and each retinal vascular marker. (**C**) Heigh of edge (yellow line): the distance between the highest point of the neuroepithelial layer elevation and the RPE layer, Basal diameters (red line), Minimum diameter (white line). (**D**) Nasal and temporal macular hole cystoid space area (N-MHCSA, T-MHCSA) were measured by manually outlining using ImageJ software. (**E)** Macular hole area (MHA) was traced manually with ImageJ software. (**F**) Nasal and temporal total area (N-TA, T-TA) were manually delineated and measured using ImageJ software, the outer boundary of the region is defined by the boundary of macular hole cystoid space area. Nasal and temporal macular hole retina area (N-MHRA, T-MHRA) were regional organizations indicated by arrows. (**G**) The area of the nasal edge (T) of MH was greater than that of the temporal edge (N), N/T > 1. (**H**) The area of the temporal edge (T) of MH was greater than that of the nasal edge (N), N/T < 1.

In this retrospective study, 39 cases involving 42 eyes were analyzed (Table 1). The cohort consisted of 5 males and 34 females, averaging 64.86 ± 6.07 years in age. The preoperative Log MAR Best Corrected Visual Acuity (BCVA) was 0.87 (0.70, 1.52). Out of these, 14 patients underwent combined cataract surgery during the operation. The preoperative measurements included a basal diameter of 815 ± 253 μm and a minimum diameter of 491 ± 184 μm. The height of the nasal and temporal edges of the hole was 415 ± 70 μm and 414 ± 70 μm respectively (Fig. 1C). The nasal traction area (N-TA) measured at approximately (10.17 ± 3.35) × 10^5^ μm^2^, and the temporal traction area (T-TA) at (10.43 ± 3.81) × 10^5^ μm^2^ (Fig. 1F). Nasal macular hole cystoid space area (N-MHCSA) was (1.85 ± 1.29) × 10^5^ μm^2^, and temporal macular hole cystoid space area (T-MHCSA) was (1.65 ± 1.29) × 10^5^ μm^2^ (Fig. 1D). Nasal macular hole retinal area (N-MHRA) was (8.32 ± 2.86) × 10^5^ μm^2^ and temporal macular hole retinal area (T-MHRA) was (8.78 ± 3.16) × 10^5^ μm^2^ (Fig. 1F). The macular hole area (MHA) was recorded at (6.39 ± 2.64) × 10^5^ μm^2^ (Fig. 1E), and the macular hole cystoid space area (MHCSA) was (3.50 ± 2.23) × 10^5^ μm^2^ with a total area (TA) of (26.99 ± 8.46) × 10^5^ μm^2^.

**Table 1:** Demographic characteristics of the subjects and microstructural characteristics of the macular hole.

Characteristics	n = 42
Male: female (patients)	5: 34
Age (years)	64.86 ± 6.07
Right: left (eyes)	13 : 29
Preoperative BCVA (Log MAR)	0.87 (0.70, 1.52)
PPV + ILM + IOL: PPV + ILM	14: 28

Linear parameters(μm)	
Basal diameter	815 ± 253
Minal diameter	491 ± 184
Height of nasal edge	415 ± 70
Height of temporal edge	414 ± 70

Area parameters(× 10^5^ μm)^2^	
TA	26.99 ± 8.46
MHA	6.39 ± 2.64
MHCSA	3.50 ± 2.23
MHRA	17.10 ± 5.70
N-TA	10.17 ± 3.35
T-TA	10.43 ± 3.81
N-MHCSA	1.85 ± 1.29
T-MHCSA	1.65 ± 1.29
N-MHRA	8.32 ± 2.86
T-MHRA	8.78 ± 3.16

BCVA, best correct visual acuity; PPV, Par plana vitrectomy; ILM, internal limiting membrane; IOL, intra-ocular lens; TA, total area; MHA, macular hole area; MHCSA, macular hole cystoid space area; MHRA, macular hole retina area; N-, nasal; T-, temporal.

### Postoperative macular displacement results

**Figure 2:**
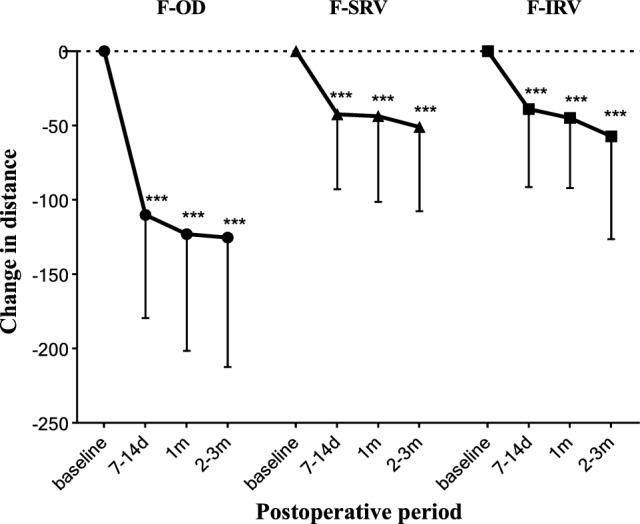
Time course of macular retina displacement after macular hole surgery. ***P < 0.001.

Following vitrectomy and ILM peeling, all macular holes were successfully closed. The preoperative distance between the optic disc and the center of the macular hole (MH-OD) was (3933.05 ± 48.00) μm. Postoperatively, however, the distance between the optic disc and the fovea (F-OD) was significantly reduced at all observed times (*P* < 0.001; Table 2), indicating a shift of the macular fovea towards the optic disc. No significant differences were found between F-OD measurements at 7–14 days post-surgery and 1 month post-surgery, or between 1 and 2–3 months post-surgery (*P* > 0.05; Table 2), suggesting that most foveal displacement occurred within the first 7–14 days postoperatively with no significant changes thereafter (Fig. 2).

**Table 2:** Postoperative changes in the distance between macular fovea and vascular marker points.

Period	F-OD (μm)	F-SRV (μm)	F-IRV (μm)
Baseline	3933.05 ± 311.06	3998.62 ± 642.84	3530.00 ± 682.59
7-14d	3822.93 ± 285.25	3956.12 ± 643.45	3491.07 ± 676.64
1 m	3811.19 ± 291.89	3954.88 ± 643.91	3485.17 ± 684.24
2-3 m	3803.12 ± 287.92	3947.67 ± 648.89	3472.86 ± 701.60
*P*-value	< 0.001	< 0.001	< 0.001

F-OD, distance between the fovea and optic disc; F-SRV, distance between fovea and superior retina vascular bifurcation; F-IRV, distance between the fovea and inferior retina vascular bifurcation.

Additionally, preoperative and postoperative distances between the macular center and the vascular bifurcation points of the superior temporal (F-SRV) and inferior temporal (F-IRV) vascular arches were analyzed. Both F-SRV and F-IRV distances at each postoperative time point were significantly shorter compared to preoperative measurements (*P* < 0.001, Table 2), with no significant differences between the F-SRV and F-IRV distances postoperatively (*P* > 0.05, Table 2). Consequently, the F-SRV and F-IRV distances did not exhibit significant changes beyond 7–14 days postoperatively. These findings indicate that there was a positional shift towards the optic disc following IMH surgery. This suggests that the displacement of fovea towards the optic disc also occurred primarily within the early postoperative period (7–14 days), stabilizing afterwards (Fig. 2).

### Correlation between linear and area parameters

The relationship between each preoperative linear parameter of the macular hole and the area parameter was examined. There were correlations between the following: (1) the preoperative basal diameter of the macular hole and the preoperative MHA (*r*_*s*_ = 0.884; *P* < 0.001), as well as a correlation with MHCSA (*r*_*s*_ = 0.432; *P* < 0.01), and a correlation with TA (*r*_*s*_ = 0.685; *P* < 0.001). (2) The preoperative minimum diameter of the macular hole and MHA (*r*_*s*_ = 0.833; *P* < 0.001), but not with MHCSA (*P* > 0.05). There was also a correlation with TA (*r*_*s*_ = 0.608; *P* < 0.001). (3) The height of the nasal edge of the preoperative macular hole and MHA (*r*_*s*_ = 0.433; *P* < 0.01), as well as with MHCSA (*r*_*s*_ = 0.682; *P* < 0.001), and with TA (*r*_*s*_ = 0.560; *P* < 0.001). (4) The height of the temporal edge and with MHA (*r*_*s*_ = 0.451; *P* < 0.01), along with a correlation with MHCSA (*r*_*s*_ = 0.679; *P* < 0.001), and with TA (*r*_*s*_ = 0.551; *P* < 0.001).

### Correlation between preoperative index and displacement

As previously mentioned, the degree of fovea displacement was no longer significant after two weeks postoperatively. Therefore, we used the displacement(%) of the fovea at 7–14 days postoperatively to calculate the degree of displacement. We then analyzed the correlation between the preoperative parameter of MH and displacement(%) (Table 3). The results were as follows: (1) There was a correlation between the basal diameter and the displacement(%) (*r*_*s*_ = 0.405, *P* = 0.008). (2) There was no correlation between the minimum diameter and the displacement(%) (*P* > 0.05). (3) There was no correlation between the height of macular hole and the displacement(%) (All *P* > 0.05). Based on a previous analysis of the correlations among preoperative parameters, which revealed a correlation between the preoperative basal diameter and each area parameter, we further analyzed the correlations between MHA, MHRA, MHCSA, and TA and the displacement(%). We used Partial Correlations Analysis to control for the basal diameter variable and found no correlation between these parameters and the degree of postoperative displacement (*P* > 0.05).

**Table 3:** Results of correlation analysis of preoperative macular hole parameters with the degree of postoperative displacement.

Variables	*r*_*s*_	*P*-value
Basal diameter (μm)	0.405	0.008
Minimal diameter (μm)	0.260	0.096
Height of nasal edge (μm)	0.050	0.755
Height of temporal edge (μm)	0.269	0.085
MHA (μm)^2^	0.163	0.307
MHRA (μm)^2^	0.284	0.072
MHCSA (μm)^2^	− 0.051	0.751
TA (μm)^2^	0.252	0.112

MHA, macular hole area; MHCSA, macular hole cystoid space area; MHRA, macular hole retina area; TA, total area.

**Figure 3:**
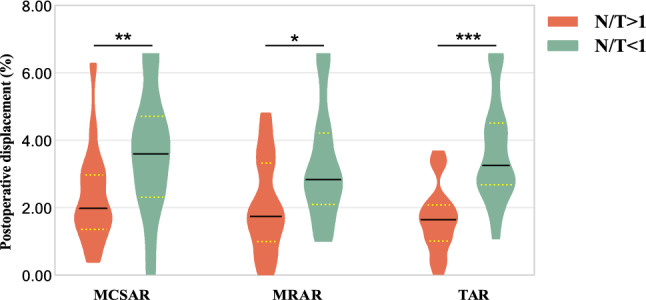
Effect of the ratio of nasal and temporal area parameter on postoperative fovea displacement. MHCSAR, Macular hole cystoid space area ratio; MHRAR, macular hole retina area ratio; TAR, total area ratio; N/T > 1, nasal area greater than temporal area; N/T < 1, nasal area less than MHRAR, macular hole retina area ratio; TAR, total area ratio; N/T > 1, nasal area greater than temporal area; N/T < 1, nasal area less than temporal area; N/T > 1, nasal area greater than temporal area; N/T < 1, nasal area less than temporal area. *P < 0.05, **P < 0.01, ***P < *0*.001.

Additionally, we measured the cystoid space area on both the nasal and temporal sides of the macular hole and calculated the retinal tissue area for each side respectively. As both sides were pulled differently, we used the ratio of area parameters to determine which side, nasal or temporal, was more significantly affected preoperatively. We divided into two groups based on whether the area ratio (MHCSAR, MHRAR, TAR) of the nasal and temporal sides affected by traction was greater or less than 1 (Fig. 1G, H). These groups were termed the group with severe involvement of the nasal side due to traction (N/T > 1) and the group with severe involvement of the temporal side due to traction (N/T < 1). The MHCSAR > 1 group had 26 eyes (62%), while the MHCSAR < 1 group had 16 eyes (38%). The MHRAR > 1 group had 17 eyes (40%), and the MHRAR < 1 group had 25 eyes (60%). Furthermore, the TAR > 1 group had 19 eyes (45%), and the TAR < 1 group had 23 eyes (55%). The results showed significant differences in the degree of postoperative displacement between the two groups (*P*_MHCSAR_ < 0.001, *P*_MHRAR_ < 0.01, *P*_TAR_ < 0.05, Table 4), with greater degrees of postoperative displacement in the MHCSAR < 1, MHRAR < 1, and MTAR < 1 groups (Fig. 3).

**Table 4:** Effect of preoperative ratio of area parameters on both sides of MH on postoperative displacement of the fovea.

Group	Postoperative displacement [*M* (*Q1, Q2*), %]	*Z*-value	*P*-value
MHCSAR > 1	1.98 (1.35, 2.97)	− 2.745	0.006
MHCSAR < 1	3.60 (2.31, 4.72)		
MHRAR > 1	1.75 (0.99, 3.32)	− 2.268	0.023
MHRAR < 1	2.84 (2.10, 4.22)		
TAR > 1	1.64 (1.01, 2.09)	− 4.081	< 0.001
TAR < 1	3.26 (2.68, 4.51)		

MHCSAR, macular hole cystoid space area ratio; MHRAR, macular hole retina area ratio; TAR, total area ratio.

Furthermore, a linear regression analysis was conducted to explore all factors that might influence the degree of postoperative displacement. The effects of the basal diameter, preoperative MHRAR, and preoperative TAR on the degree of postoperative displacement were statistically significant. The results indicated that: (1) the more significant the preoperative basal diameter, the greater the degree of postoperative displacement (*P* < 0.05, *R*^*2*^ = 0.145, standardized regression coefficient = 0.381). (2) The smaller the preoperative TAR, the greater the displacement(%) (*P* < 0.01, *R*^*2*^ = 0.209, standardized regression coefficient = − 0.457). (3) The smaller the preoperative MAR, the greater the degree of postoperative displacement (*P* < 0.05, *R*^*2*^ = 0.143, standardized regression coefficient = − 0.379). (4) There was no statistically significant effect of MHCSAR preoperatively on the displacement(%) (*P* = 0.08). Therefore, the results suggest that the greater the degree of preoperative pulling influence on the temporal side, the greater the degree of postoperative displacement of the central recess toward the optic disc.

### Correlation between degree of displacement and postoperative visual acuity

Log MAR BCVA in 42 IMH eyes was 0.87 (0.70, 1.52) preoperatively, 0.70 (0.60, 1.08) at the 7–14 day postoperative follow-up, 0.65 (0.52, 0.80) at the 1-month postoperative follow-up, and 0.60 (0.40, 0.70), and the results showed that visual acuity improved at all postoperative time points at follow-up compared with preoperative (all *P* < 0.001). In addition, we found no correlation between macular center displacement (%) and Log MAR BCVA at postoperative follow-up at any of the postoperative time points (all *P* > 0.05).

## Discussion

This retrospective study analyzed 42 eyes with idiopathic macular holes (IMH) treated with pars plana vitrectomy (PPV) and internal limiting membrane (ILM) peeling, specifically selecting cases filled with sterilized air intraoperatively to eliminate potential effects from vitreous cavity-filling substances^10^. The study found that postoperative distances from the fovea to the optic disc (F-OD), superior temporal vascular arch (F-SRV), and inferior temporal vascular arch (F-IRV) were consistently reduced, indicating a central movement of the macula towards the optic disc after IMH closure. This displacement is related to the retina’s increased compliance in the macular region after ILM peeling^10,15^. In normal conditions, the ILM is not present on the surface of the optic disc. The retina on the nasal side of the macula is fixed to the optic disc, and the ILM acts as a tissue that provides support and stiffness to the retina. It balances the fixation tugging force of the optic disc on the nasal side of the retina^7,16^. After the posterior vitreous cortex's traction and the ILM's binding are released, the temporal retina in the macular region becomes more flexible, and it tends to have greater mobility compared to the nasal retina^9^. This increased flexibility and mobility of the temporal retina contribute to the observed displacement of the macular center toward the optic disc side^9^. A larger preoperative basal diameter requires more extensive coverage of the basal area and entails greater temporal retina movement during the closure maneuver^9^. This may also explain why the size of the preoperative basal diameter can influence the degree of displacement after surgery. Kawano et al.’s study comparing eyes with spontaneous idiopathic macular hole (IMH) closure and those with internal limiting membrane (ILM) peeling revealed that macular displacement didn’t occur in spontaneously closed cases. This suggests that ILM removal is a key factor in initiating macular displacement^7^. Similarly, in a study by Yoshikawa et al.^17^ involving 72 eyes with and without ILM removal for diabetic macular edema, it was observed that the macula-to-disc distance was smaller in eyes with ILM removal compared to those without. Therefore, researchers supposed that the retina has a potential contractile property, primarily stemming from the contraction of the retinal nerve fiber layer following the loss of support provided by the ILM^18^. The contraction of the retinal nerve fiber layer is microscopically attributed to changes in the macular microenvironment after ILM removal or to unknown factors that cause microtubule depolymerization, leading to axonal contraction^19^. In conclusion, foveal displacement towards the nasal side following idiopathic macular hole (IMH) treatment is a result of rebalanced internal limiting membrane (ILM) binding forces on the macular retinal surface post-ILM release. On the nasal side, the retina experiences a dual force: centripetal contraction aiding hole closure and the optic disc’s constant pull. Conversely, on the temporal side, both the centripetal contraction and retinal nerve fiber layer contraction propel the retina towards the optic disc. This asymmetry in retinal contraction forces causes more extensive movement of the temporal retina compared to the nasal side, culminating in the overall macular shift towards the optic disc^20^. Moreover, our study found that changes of F-OD, F-SRV and F-IRV, become statistically insignificant after the first two weeks post-surgery. This indicates a stabilization of the macular displacement, aligning with previous research that suggests the displacement occurs early post-ILM peeling^9,10^. Given that the displacement and the closure of the macular hole both predominantly occur within the same early postoperative period, it's hypothesized that there is a correlation between these two events. The timing suggests that the mechanisms driving the foveal displacement towards the nasal side might be intrinsically linked to the processes that facilitate the closure of the macular hole. This hypothesis warrants further investigation to confirm the relationship and to understand the underlying biological mechanisms.

In this study, the focus was on analyzing the impact of various preoperative parameters of the macular hole on the extent of postoperative foveal displacement. Uniquely, this research also explored the correlation between the preoperative tractional force areas on both the nasal and temporal sides of the macular hole and the subsequent degree of foveal displacement. The findings revealed that a larger preoperative tractional force on the temporal side of the hole edge was associated with a more significant displacement of the central fovea towards the optic disc after the hole had closed. In this study, the evaluation of traction force exerted on the margins of the macular hole was based on the measurement of the involved area on both the nasal and temporal sides. The lateral border of this area was demarcated by the edge of the cystic cavity, meaning the size of the cystic cavity essentially dictated the calculation of the involved area. It's acknowledged that vitreous macula traction plays a pivotal role in the formation of macular holes, inducing the development of intraretinal cystic cavities as a response to pulling forces^21,22^. Notably, a vast majority of idiopathic macular hole (IMH) cases feature these fluid-filled cavities around the hole, caused by a decrease in interstitial fluid pressure in the retina from vitreomacular traction, leading to fluid leakage^23,24^. As the edges of the hole detach from the retinal pigment epithelium (RPE) layer, fluid accumulates in the interstitial space, which the RPE can’t efficiently reabsorb. Therefore, assessing the area of involvement provides a rough estimation of the magnitude of preoperative pulling forces around the hole. Traditional linear parameters like basal diameter and hole height reflect the horizontal and anterior–posterior traction forces on the hole but may only indicate traction in a single direction Conversely, calculating the area of involvement offers a more comprehensive understanding of the forces around the hole. Moreover, manually defining a range for this calculation adheres to the curved surface characteristics of the hole margins^13^. More significant area parameters, indicative of more vital pulling forces, are associated with larger basal diameters, higher edge of the hole, and relatively lower preoperative visual acuity, consistent with previous findings in the report^24–27^. These associations reinforce the concept that area parameters are more reliable indicators of the extent of preoperative traction near the macular hole (MH). Additionally, more preoperative traction has been associated with a higher probability of successful hole closure after surgical intervention. This is possibly due to the fact that the release of greater traction forces allows for more mobility in the macular margins, facilitating the hole’s closure^13^. Baba et al.^28^ found a significant reduction of avascular area in the center of the macula after IMH, which means that the closure process is accompanied by a centripetal convergence of the retina^29,30^. However, after the greater traction force is released, the margins of the macula on both sides have greater mobility and are therefore, more likely to close. Also, as mentioned earlier, the macular center displaces toward the optic disc after the closure of the hole because the temporal edge moves a larger area to cover the base. Therefore, eyes with IMH and greater preoperative involvement of the temporal edge experience greater mobility on the temporal side after detachment. This suggests that the temporal retina can move more extensively to facilitate closure, leading to a more significant displacement of the fovea toward the optic disc. Previous researchers has inferred that displacement may be a beneficial process for the closure of MH^7^, and our findings degree with this idea, highlighting that the more mobile temporal edge likely plays a significant role in closing the hole and causing a pronounced shift in the position of the central recess after surgery.

The study found no correlation between the degree of displacement and the recovery of postoperative visual acuity, which is consistent with the findings of previous studies^7,31^. The displacement phenomenon and the observed previously separation and swelling of optic nerve fibers resulting from ILM peeling are now regarded as subclinical changes. Some patients continue to experience metamorphopsia, or visual distortion, post-surgery for IMH. Research by Kim et al.^31^ and Park et al.^18^ suggests a correlation between asymmetric changes in the fovea, displacement, and postoperative visual distortion. While displacement doesn’t hinder visual recovery or macular hole closure, it may affect the precise transmission of visual signals by altering the tertiary neuronal connections within the retina. This disturbance doesn’t sever or disrupt neuronal connections, which would significantly impact visual recovery, but it may exert a tangential force on the retinal layers^31,32^. The study, however, did not specifically assess metamorphopsia symptoms or fully explore the potential impacts of displacement on visual function. Therefore, it is crucial to note that the traditional procedure of ILM peeling, involving the disruption of Müller cell peduncles, inevitably leads to some retinal damage in the peeled area^33,34^. The investigation of the mechanisms that cause damage will help us to understand the potential damage of ILM movement, to a certain extent, and assist surgeons in making more informed decisions regarding the surgical approach and its timing.

Our study has several limitations. Firstly, it is a retrospective study, despite our best efforts to screen enrolled patients, however, it is susceptible to biases related to surgical factors, such as the dimension and direction of ILM peeling. Secondly, the study experienced some patient loss due to COVID-19. Thirdly, the data related to displacement and other parameters were manually measured from OCT images, which introduces the possibility of measurement errors affecting the results. Fourthly, patient variations in the postoperative prone position and time could impact the results. We hope that future research will address these limitations by conducting more extensive prospective studies with larger sample sizes and increased detail.

## Methods

### Ethical approval

This retrospective study adhered to the principles outlined in the Declaration of Helsinki. Approval was granted by the Ethical Review Organization of the Second Hospital of Jilin University (Approval No. 2022-020), and informed consent was obtained from all participating subjects.

### Study subjects

This study focused on patients with IMH treated at the Eye Center of the Second Hospital of Jilin University between January 2020 and April 2023. All procedures were conducted by the same surgeon, using PPV combined with ILM peeling, sometimes accompanied by cataract extraction. The patients underwent thorough preoperative and postoperative ophthalmologic examinations, including slit lamp microscopy, intraocular pressure measurement, best corrected visual acuity testing, and OCT. Inclusion criteria: (1) Patients who underwent PPV and ILM peeling, with or without concurrent cataract extraction for IMH. (2) Traditional ILM peeling was performed, with a peeling diameter of 2–3 disc diameters. (3) The vitreous cavity was filled with sterilized air. (4) Successful closure of the IMH after the initial surgery. (5) A minimum of 2 months follow-up period. (6) Stage 2, 3, or 4 full-thickness IMH identifiable by OCT. Exclusion criteria: (1) Patients with myopic > 6.00D and hyperopia > 3.00D. (2) A history of glaucoma, optic disc or optic nerve diseases, other vitreoretinal diseases (e.g., diabetic retinopathy, retinal detachment, retinitis pigmentosa, uveitis, or other macular diseases), ocular trauma, or prior vitrectomy surgery. (3) Incomplete medical records, loss to follow-up, or poor imaging quality during examination.

### Surgical procedure

All patients underwent a standard 25-gauge three-channel vitrectomy. In cases where severe cataracts hindered intraocular manipulation, intraocular lens implantation was performed. The vitreous cavity received an injection of triamcinolone acetonide to facilitate staining of the ILM. The residual posterior vitreous cortical membrane and the macular ILM were carefully peeled away using ILM forceps, extending approximately 2–3 disc diameters. Finally, the vitreous cavity was filled with sterile air.

### Data collection

The best-corrected visual acuity and OCT images of the patients were collected preoperatively and during postoperative follow-ups at 7–14 days, 1 month, and 2–3 months. The preoperative and postoperative follow-up OCT image reports of all patients were taken by the same OCT machine (Heidelberg). We manually measured various parameters related to the macular microstructure using the OCT machine concerning Yilmaz et al.^35^. In the horizontal scanning images, we pinpointed the locations where the retinal vessels of the superior temporal and inferior temporal areas intersected at the same position in both the preoperative and each of the postoperative follow-up OCT images. Using the OCT machine's calipers, the distances from the center of the macula to these vascular bifurcation points were measured and labeled accordingly: MH-SRV (macular hole to superior retina vessel) and MH-IRV (macular hole to inferior retina vessel) before surgery and F-SRV (fovea to superior retina vessel) and F-IRV (fovea to inferior retina vessel) after surgery. Additionally, the distance from the macular center to the origin point of the retinal vessels at the temporal optic disc rim was measured and recorded as MH-OD preoperatively and F-OD postoperatively (Fig. 1A, B). When studying the factors that impact the extent of displacement, the degree of macular displacement was quantified as a percentage, denoted as “displacement (%),” and was calculated by finding the difference between MH-OD and F-OD, divided by MH-OD. In the vertical scanning OCT images, the preoperative basal diameter (μm) and minimum diameter (μm) of the hole and the height of edge (μm) on the nasal and temporal sides were also measured (Fig. 1C). Moreover, the assessment of area parameters was performed utilizing the ImageJ image processing software in a systematic manner. The preoperative SD-OCT images were imported into the software, guaranteeing consistent proportions in both vertical and horizontal directions and uniform pixel (1280 pixels × 860 pixels). The polygonal tool, which was made available by the software, was utilized to outline the region of interest within the image and subsequently compute its measurements. We determined the preoperative nasal macular hole cystoid space area and temporal macular hole cystoid space area. (N-MHCSA and T-MHCSA, μm^2^, Fig. 1D), the macular hole area (MHA, μm^2^, Fig. 1E), the nasal and temporal total area of the macular hole influenced by traction (N-TA, T-TA, μm^2^, Fig. 1F). These measurements were obtained by manually delineating the regions of interest using the ImageJ image-processing software, and the following metrics were calculated from the measured values:

1. Nasal macular hole retinal area (N-MHRA) = N-TA−N-MHCSA (Temporal side is the same as the nasal side)
2. Total area influenced by traction (TA) = N-TA + MHA + T-TA
3. The ratio of macular hole cystoid space area (MHCSAR) = N-MHCSA**/**T-MHCSA
4. The ratio of macular hole retinal area (MHRAR) = N-MHRA**/**T-MHRA
5. The ratio of the total area of the hole influenced by traction (TAR) = N-TA**/**T-TA

### Statistical analysis

Statistical analysis was carried out using SPSS version 26.0. We presented the results as mean ± standard deviation for measures that followed a normal distribution. Non-normally distributed measures were represented as [M (Q1, Q2)], and count data were expressed as n (%). We used the Shapiro–Wilk test to assess the normality of continuous variables. To compare differences across various time points, we employed a Repetitive Measure Analysis of Variance, Two-by-two data comparisons were corrected using the Bonferroni method. Mann–Whitney U test was used to compare data between different groups. Pearson's correlation analysis was performed for variables that exhibited a normal distribution, with the correlation coefficient denoted as '*r*.' For variables that did not meet the criteria for normal distribution, Spearman’s correlation analysis was used to assess statistical correlations, and the correlation coefficient was expressed as ‘*r*_*s*_.’ We compared the absolute value of the correlation coefficient, where an absolute value ≤ 0.3 indicated no correlation, 0.3–0.5 indicated a low correlation, 0.5–0.8 indicated a moderate correlation, and 0.8–1 indicated a high correlation. Possible correlations were further analyzed using linear regression, and differences were considered statistically significant if the *P*-value was less than 0.05.

## Data Availability

All data generated or analysed during this study are included in this article (and its Supplementary Information files).
